# Supplementary material for: A unique serum IgG glycosylation signature predicts development of Crohn’s disease and is associated with pathogenic antibodies to mannose glycan
Source: Nat Immunol. 2024 Jul 30;25(9):1692–703. doi: 10.1038/s41590-024-01916-8 (PMC11362009; doi:10.1038/s41590-024-01916-8)

nature immunology

Supplementary information

https://doi.org/10.1038/s41590-024-01916-8

A unique serum IgG glycosylation signature predicts development of Crohn's disease and is associated with pathogenic antibodies to mannose glycan

In the format provided by the authors and unedited

SUPPLEMENTARY METHODS

Glycopeptide preparation

To isolated human and murine IgGs, 0.2 µg of TPCK-treated trypsin (Promega, Madison, WI) was added, followed by an overnight incubation at 37°C. Tryptic digests were purified using a solid phase extraction (SPE) on C18 beads (Chromabond, Macherey-Nagel, Duren, Germany). 10 ug of C18 beads was applied to each well of an OF1100 96-well polypropylene filter plate (Orochem, Naperville, IL). The RP stationary phase was activated 3 times with 200 µl of 80 % ACN and conditioned with 3 times with 200 µl 0.1 % TFA (Sigma- Aldrich/Merck, Darmstadt, Germany). The IgG digests were diluted with 0.1 % TFA (Sigma-Aldrich/Merck) loaded onto the C18 beads and washed 3 times 200 µl 0.1 % TFA. The entire procedure was performed on a vacuum manifold under pressure reduction of 2 inches of mercury. IgG glycopeptides were eluted into a PCR 96 well plate with 200 µl of 20 % ACN by centrifugation at 105 g for 5 min. Eluates were dried by vacuum centrifugation and stored at - 20 °C until analysis by MS.

Human IgA enrichment and digestion

20 µL of plasma samples were transferred to wells of PCR plate and diluted with 110 µL of 1xPBS, pH 7.4. Diluted plasma samples were transferred into corresponding wells of Orochem filter plate containing 40 µL of IgA beads slurry (Thermo Scientific, IgA Affinity Matrix). Following 1 hour of incubation and several steps of washing with 1x PBS and MQ water, captured IgA was eluted with 100 mM formic acid. The collected eluate was dried in a speedvac at 60°C for 2.5 hours and resuspended in 25 mM ammonium bicarbonate for the subsequent reduction and alkylation with 200 mM DTT (Sigma-Aldrich) and 400 mM IAA (Sigma-Aldrich). Finally, to the reduced and alkylated sample, 1 µL of 0.2 µg/ µL TPCK-treated trypsin stock (Promega, Madison, WI) was added, followed by an overnight incubation at 37°C. Samples were stored at -20°C until the analysis by LC-MS.

nanoLC-ESI-MS of human IgG, human IgA and murine IgG N-glycopeptides

Purified tryptic human IgG, human IgA and murine IgG glycopeptides were analyzed on Waters ACQUITY M Class UPLC system (Waters, Milford, MA), consisting of binary pump, auxiliary pump, trap valve manager and autosampler maintained at 10 °C with column compartment set to 30°C. 10 µL of sample was applied to a PepMap 100 C18 (5 mm × 300 µm i.d., 5µm, Thermo) SPE trap column conditioned with 0.1 % TFA (mobile phase A) for 1 min at 40 µl/min. For human IgGs: after sample loading, the trap column was switched in-line with the gradient and C18 nano-481 LC column (150 mm x 100 µm i.d., 2.7 µm HALO fused core particles; Advanced Materials Technology, Wilmington, DE) for 9.5 minutes. Trap column was cleaned with two full loop injections containing 20 µl of 95 % ACN and 50% ACN in IPA respectively. C18 nano-LC column was equilibrated with 100 % mobile phase A (0.1% TFA), for 2 minutes. IgG glycopeptides were reconstituted in 100 µl of ultrapure water before injection. Separation was achieved at 1 ml/min using the following gradient of mobile phase A and mobile phase B (80 % ACN and 20 % 0.1 % TFA respectively): 0.5 min 12 % B, 0.5 – 4 min 12 % B - 17 % B, 4 - 5 min 17 % B. The ACQUITY M-Class system was coupled with Compact mass spectrometer (Bruker Daltonics, Bremen, Germany) equipped with Captive Spray ion source and nano Booster for introduction of acetonitrile vapor into the source. Nitrogen was used as a drying gas (4 l/min) and nebulizer (0.4 bars). Quadrupole and collision energies were set to 4 eV. Spectra were recorded from m/z 800 to 2000 with 2 averaged scans at a frequency of 0.5 Hz. Acquity M-class UPLC system was operated under MassLynx software 4.1 while Bruker Compact Q-TOF-MS was operated under HyStar software version 4.1. For human IgA, and murine IgGs: after sample loading, the trap column was switched in-line with the gradient and C18 nano LC column (150 mm x 100 µm i.d., 2.6 µm SunShell core shell particles; Chromanik Technologies Inc.) for 9 minutes. Trap column was cleaned with two full loop injections containing 20 µl of 95 % ACN and 50% ACN in IPA respectively. C18 nano-LC column was equilibrated with 100 % mobile phase A (0.1% TFA), for 2 minutes. IgG glycopeptides were reconstituted in 30 µl of ultrapure water before injection. Separation was achieved at 1 ml/min using the following gradient of mobile phase A and mobile phase B (80 % ACN and 20 % 0.1 % TFA respectively): 1 – 1.5 min 0 % B – 18.5 % B, 1.5 - 5 min 18.5 % B – 26 % B, 5 - 9 min 26 % B. The ACQUITY M-Class system was coupled with Compact mass spectrometer (Bruker Daltonics, Bremen, Germany) equipped with Captive Spray ion source and nano Booster for

introduction of acetonitrile vapor into the source. Nitrogen was used as a drying gas (4 l/min) and nebulizer (0.2 bars). Quadrupole and collision energies were set to 4 eV. Spectra were recorded from m/z 600 to 2500 with 2 averaged scans at a frequency of 0.5 Hz. Acquity M-class UPLC system was operated under MassLynx software 4.1 while Bruker Compact Q-TOF-MS was operated under HyStar software version 4.1. The detected glycopeptides are presented in **Supplementary Table 2**, and in accordance with previous reports[1, 2, 3].

IgG aggregation assay

To assess the degree of IgG aggregation, a subset of IgG samples from healthy controls and CD patients (preclinical phase, at diagnosis, or full-blown disease) were analyzed using a protein aggregation assay kit (Abcam), according to manufacturer's instructions. Besides the standards from the kit, a commercial IgG antibody was incubated at 65ºC for 45 minutes to aggregate (positive control). Fluorescence was measured at Ex/Em 440/500 nm and compared with the standard curve of the kit.

Isolation of Natural Killer (NK) cells

NK cells were purified by negative selection from freshly PBMCs using EasySep™ Human NK Cell Isolation Kit, according to the manufacturer's instructions (Stem Cell Technologies). The purity (>94%) of NK cells was determined by flow cytometry analysis on FACSCanto™ II system (BD Biosciences, San Jose, CA). Monoclonal antibodies used for staining of NK cells are shown in **Supplementary Table 6**. Cells were cryopreserved in freeze medium (90% of fetal bovine serum (FBS) and 10% of dimethyl sulfoxide (DMSO)) at -80°C. After defrosting, cryo-preserved PBMCs and cryo-preserved NK cells were maintained in complete culture medium, RPMI 1640 Medium, GlutaMAX™ Supplement, HEPES (Thermo Fisher Scientific), supplemented with 10% of FBS, 1% penicillin/streptomycin and 0,1% gentamicin overnight at 37°C in a humidified incubator with 5% of CO_2.

IgG-dependent NK cell degranulation

Purified NK cells were suspended in complete RPMI medium supplemented with IL-2 (100 U/mL, Pepotrech) and plated in wells previously coated with mannan, β-glucan or di-GlcNAc (as described above for DCs), at $1x10^5$ cells/well in the presence of anti-human CD107a (LAMP-1; **Supplementary Table 6**). Following 1 hour, 10 µg/ml of Brefeldin A (Sigma–Aldrich) and GolgiStop™ Protein Transport Inhibitor were added and incubated 5 hours in similar conditions. After incubation, cells were stained for viability, as well as for surface markers (**Supplementary Table 6**). Cells were fixed and permeabilized with Transcription Factor Staining Buffer Set (eBioscience), followed by intracellular staining (**Supplementary Table 6**). Samples were acquired by flow cytometry on a FACSCanto™ II system (BD Biosciences) using the FACSDiva™ software (BD), and data were analyzed in FlowJo version 10.5.3 (Tree Star, Inc., Ashland, OR), using gating strategy shown in **Supplementary Fig. 9b**.

C1q (complement) binding assay

96-well Nunc® MaxiSorp™ high-binding plates were pre-coated with mannan as previously described. A blocking step with 5% of BSA was performed. 6 µg/mL of total IgGs were added to plates previously coated with mannan (10 µg/mL), for 90 minutes at 37°C in a humidified incubator with 5% of CO_2. After three washing steps with PBS 3% BSA 0.05% Tween20, complement component C1q was added (10 µg/mL) for 1 hour at room temperature. After washing (3x), HRP sheep anti-human C1q was added for incubation for 1 hour at room temperature. Plate was washed four times (as previously), and 3,3',5,5'-tetramethylbenzidine (substrate solution) was added for 15 min. Reaction was stopped with 1 N H_2SO_4 and absorbance was measured at 450 nm.

1. Momčilović, A. *et al.* Simultaneous Immunoglobulin A and G Glycopeptide Profiling for High-Throughput Applications. *Anal Chem* **92**, 4518-4526 (2020).

2. Selman, M.H. *et al.* Fc specific IgG glycosylation profiling by robust nano-reverse phase HPLC-MS using a sheath-flow ESI sprayer interface. *J Proteomics* **75**, 1318-1329 (2012).

3. Zaytseva, O.O. *et al.* Fc-Linked IgG N-Glycosylation in FcγR Knock-Out Mice. *Front Cell Dev Biol* **8**, 67 (2020).

SUPPLEMENTARY TABLES

Supplementary Table 1. Demographic and clinical characteristics of the samples from the PREDICTS cohort.

		Patients with Crohn's disease	Patients with ulcerative colitis	Healthy control individuals
**Number of individuals**		251	249	250
**Number of samples**		1002	992	997
**Age**[a] years; mean ± SD		31.70 ± 6.53	29.77 ± 5.78	29.67 ± 5.65
**Sex** n (%)	Female	184 (18.36)	102 (10.28)	83 (8.32)
	Male	818 (81.64)	890 (89.72)	914 (91.68)

[a]Age at sample A (healthy individuals) or diagnosis (inflammatory bowel disease).

Supplementary Table 2. Glycan traits of IgG and IgA characterized in serum samples.

**IgG glycan traits**		
**Glycan Trait**	**Galactosylation profile**	**Additional glycan structures**
H3N4	agalactosylated (G0)	-
H3N4F1		fucosylated
H3N5F1		fucosylated; bisecting
H3N5		bisecting
H4N4	monogalactosylated (G1)	-
H4N4F1		fucosylated
H4N4F1S1		fucosylated; sialylated
H4N5F1		fucosylated; bisecting
H4N5F1S1		fucosylated; sialylated; bisecting
H4N5		bisecting
H5N4	digalactosylated (G2)	-
H5N4F1		fucosylated
H5N4F1S1		fucosylated; sialylated
H5N4S1		sialylated
H5N5F1		fucosylated; bisecting
H5N5F1S1		fucosylated; sialylated; bisecting

**IgA glycan traits**			
**Glycan Trait**	**Galactosylation profile**	**Additional glycan structures**	**Cluster[a]**
H3N5	agalactosylated (G0)	bisecting	LSL
H4N4S1	monogalactosylated (G1)	sialylated	LSL
H5N5S2F1	digalactosylated (G2)	fucosylated; sialylated; bisecting	LAGcb
H5N4S1		sialylated	LAGy
H5N4S1F1		fucosylated; sialylated	LAGy
H5N4S2		sialylated	LAGy
H5N5S2F1		fucosylated; sialylated; bisecting	LAGy
H5N2		high-mannose	LSL

[a]Cluster names for IgA are referring to the first three letters of the peptide sequence.

Supplementary Table 3. Demographic and clinical characteristics of the samples from established CD patients.

		**Patients with established Crohn's disease**
**Number of patients**		10
**Number of samples**		33
**Age** years; mean ± SD		53.50 ± 15.98
**Time since diagnosis** years; mean ± SD		19.04 ± 8.65
**Sex** n (%)	Female	3 (30)
	Male	7 (70)
**Clinical symptoms** *samples* n (%)	Remission	20 (60.61)
	Activity	13 (39.39)
**Therapy** *samples* n (%)	Infliximab	16 (48.48)
	Adalimumab	16 (48.48)
	Vedolizumab	1 (3.03)

Supplementary Table 4. Demographic and clinical characteristics of the samples from inaugural CD patients, first-degree relatives (FDR) of CD patients and healthy controls.

		Patients with Crohn's disease	First-degree relatives	Healthy control individuals
**Number of individuals**		6	7	6
**Number of samples**		6	7	6
**Age**[a] years; mean ± SD		31.83 ± 12.97	25.71 ± 3.99	27.17 ± 1.94
**Sex** n (%)	Female	1 (16.67)	5 (71.43)	5 (83.33)
	Male	5 (83.33)	2 (28.57)	1 (16.67)

[a]Age at sample collection.

Supplementary Table 5. Disease activity index (DAI). The DAI is obtained by the average score in each category.

DAI	Weight loss (%)	Stool consistency	Blood
0	0	WF	No blood
1	1-5	WF/P	-
2	6-10	P	Vestiges in the swab
3	11-15	P/L	-
4	16-20	L	Blood in the swab
5	21-25	-	-
6	>25	-	-

Supplementary Table 6. Antibodies and dyes used in flow cytometry.

Antibody	Catalog number	Company	Company
eBioscience™ Fixable Viability Dye eFluor™ 780	65-0865-18	1:2000	Invitrogen
Brilliant Violet 510 anti-human CD3, clone OKT3	317332	1:100	BioLegend
PE anti-human CD56, clone HCD56	318306	1:100	BioLegend
APC anti-human Granzyme B, clone GB14	MHGB05	1:100	Invitrogen
FITC anti-human CD107a (LAMP-1), clone H4A3	328606	1:150	BioLegend
PE anti-human CD14, clone 61D3	12-0149-42	1:100	Invitrogen
APC anti-human CD11c, clone B015	17-0128-42	1:100	Invitrogen
PE-Cyanine5 anti-human CD86, clone IT2.2	15-0869-42	1:100	Invitrogen
Rabbit anti-human CD209	AHP627	1:100	Bio-Rad
Polyclonal Swine anti rabbit Immunoglobulins/FITC	F0205	1:20	Dako
Anti-hDectin2 Affinity Purified Goat IgG	AF3114	1:100	R&D Systems
Polyclonal Rabbit anti-Goat Immunoglobulins/Biotinylated	E0466	1:100	Dako
Streptavidin PE-Cyanine7	25-4317-82	1:200	Invitrogen
PE-CF594 anti-human CD138, clone MI15	564606	1:100	BD Biosciences
PE-Cyanine5 anti-human CD38, clone HIT2	303507	1:400	BioLegend
BV605 anti-human CD3, clone 17A2	100237	1:300	BioLegend
cFR685 anti-human CD19, clone HIB19	R7-20118	1:300	Cytek
BB700 anti-human IgG, clone G18-145	742235	1:300	BD Biosciences
PE-Cyanine7 anti-human IgM, clone MHM-88	314531	1:200	BioLegend
BV605 anti-mouse CD45, clone 30-F11	103139	1:400	BioLegend
eF450 anti-mouse CD11c, clone N418	48-0114-82	1:100	Invitrogen
PE anti-mouse NKp46, clone 29A1.4	12-3351-80	1:200	Invitrogen
PerCP-Cy5.5 anti-mouse CD3	100328	1:100	BioLegend
PE-Cy5 anti-mouse MHCII, clone M5/114.15.2	15-5321-81	1:800	Invitrogen
PE-Cy7 anti-mouse CD45, clone 30-F11	25-0451-82	1:400	Invitrogen
FITC anti-mouse IFNg, clone XMG1.2	11-7311-82	1:100	Invitrogen
PE-Cy7 anti-mouse CD16/32, clone 93	101318	1:100	BioLegend
Purified anti-mouse CD16/32, clone 93	101302	1:200	BioLegend

Supplementary Table 7. Probes used for qRT-PCR analysis.

Taqman probe	Reference	Company
18S	Hs99999901_s1	Applied Biosystems
NLRP3	Hs00918082_m1	Thermo Fisher Scientific
CARD9	Hs00364485_m1	Thermo Fisher Scientific
FCGR2A	Hs01013401_g1	Thermo Fisher Scientific

Supplementary Figure 1

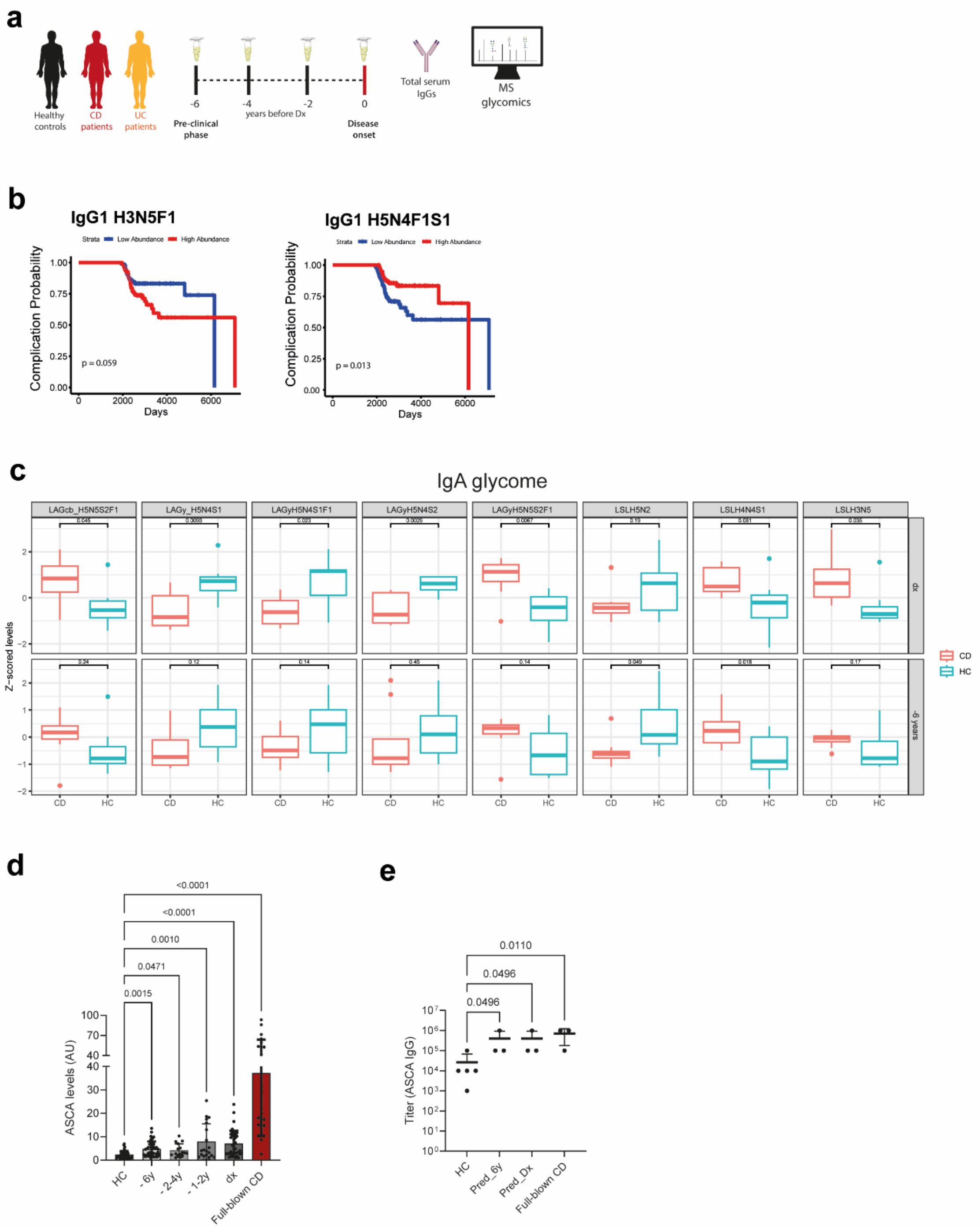

Supplementary Figure 1. Glycosylation signatures of CD patients and ASCA quantification. (a) Total IgGs from serum samples from n=251 CD patients, n=249 UC patients, and n=250 healthy controls (HC), at different time points (preclinical phase: 1-2, 4-6, and 6 years before diagnosis; and at diagnosis; matched for HC), were isolated and analyzed by mass spectrometry. **(b)** Association between different IgG glycan traits and the development of Crohn's disease complication (days to CD complication development) via Kaplan Meier. **(c)** IgA glycosylation profiles for a subset of CD patients, at pre-clinical phase (-6 years; HC, n=7; CD, n=8) and at

diagnosis (HC, n=8; CD, n=8); p-values from two-sided t-test are reported. **(d-e)** Quantification of ASCA IgG levels and titers in the serum from HC, preclinical and diagnosed CD, as well as in full-blown CD patients. In (d): HC, n=42; -6y, n=38; -2-4y, n=16; -1-2y, n=19; dx, n=40; full-blown CD, n=20. In (e), HC, n=5; Pred_6y, n=3; Pred_Dx, n=3; full-blown CD, n=3. (b: n=16-42/group; c: n=3-5/group). In (d) and (e), data presented were analyzed comparing each condition with the control (HC), by Kruskal-Wallis test with uncorrected Dunn's test and are shown as mean ± SD; p-values are shown in the graphs. Each data point represents the data from a single patient/subject (biological replicates).

Supplementary Figure 2

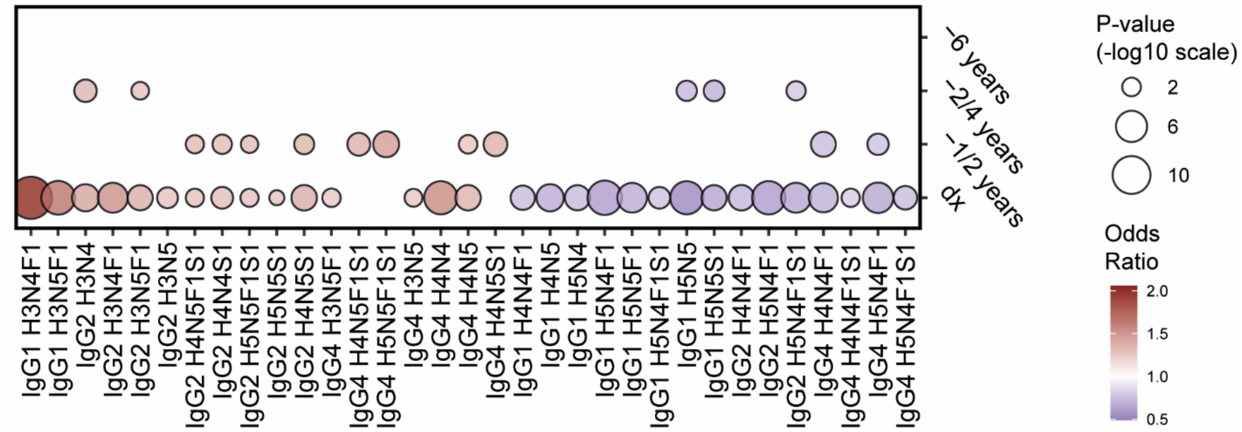

Supplementary Figure 2. Association between glycan traits measured at different time points and ulcerative colitis onset. Total IgGs from serum samples from n=249 UC patients at different time points (preclinical phase: 1-2, 4-6, and 6 years before diagnosis; and at diagnosis) were isolated and analyzed by mass spectrometry. The size of the bubble corresponds to p-value from two-sided t-test from logistic regression (-log10 scale); while the bubble color corresponds to the odds ratio. Only association significant at 10% FDR are reported.

Supplementary Figure 3

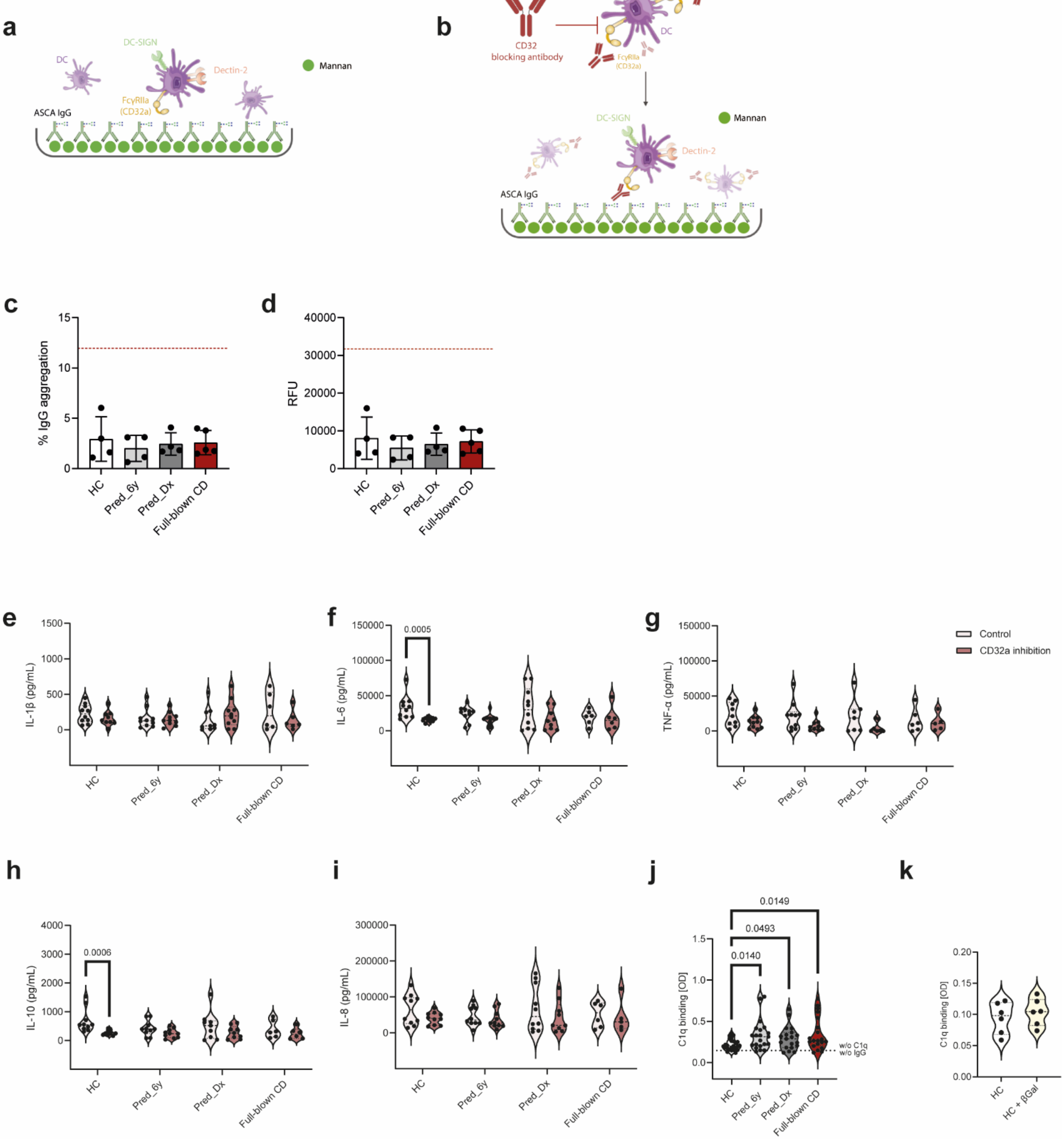

Supplementary Figure 3. Characterization of ASCA IgGs and activation properties. (a) ASCA were selected from total IgGs through binding to mannan-coated plates and then incubated with dendritic cells (DCs). **(b)** DCs with blocked FcγRIIa were also cultured with ASCA to evaluate the Fc-FcγR interaction. **(c,d)** Percentage of protein aggregation and relative fluorescence units (RFU) after IgG purification; red dashes correspond to aggregated form of a control IgG. HC (n=4), Pred_6y (n=4), Pred_Dx (n=4), Full-blown CD (established disease) (n=5). Data are presented as mean ± SD. **(e-i)** Cytokine production of DCs incubated with

ASCA from preclinical and CD samples upon blockade of FcγRIIa (CD32a). For IL-1β (e), control is composed by n=10 HC, n=10 Pred_6y, n=9 Pred_Dx and n=6 full-blown CD; CD32a inhibition condition is composed by n=9 HC, n=10 Pred_6y, n=10 Pred_Dx and n=6 full-blown CD. For IL-6 (f), control is composed by n=10 HC, n=10 Pred_6y, n=10 Pred_Dx and n=6 full-blown CD; CD32a inhibition condition is composed by n=10 HC, n=10 Pred_6y, n=9 Pred_Dx and n=6 full-blown CD. For TNF-α (g), control is composed by n=9 HC, n=10 Pred_6y, n=7 Pred_Dx and n=6 full-blown CD; CD32a inhibition condition is composed by n=10 HC, n=9 Pred_6y, n=7 Pred_Dx and n=5 full-blown CD. For IL-10 (h), control is composed by n=10 HC, n=10 Pred_6y, n=9 Pred_Dx and n=6 full-blown CD; CD32a inhibition condition is composed by n=9 HC, n=10 Pred_6y, n=9 Pred_Dx and n=6 full-blown CD. For IL-8 (i), control is composed by n=10 HC, n=10 Pred_6y, n=10 Pred_Dx and n=6 full-blown CD; CD32a inhibition condition is composed by n=10 HC, n=10 Pred_6y, n=9 Pred_Dx and n=5 full-blown CD. **(j)** C1q binding of ASCA IgGs from HC (n=25), preclinical (n=21) and CD patients (Pred_Dx, n=19; full-blown CD, n=20). **(i)** C1q binding of ASCA IgGs from HC individuals, with (n=6) or without treatment (n=6) with β-galactosidase for galactose ablation, analyzed by two-tailed Mann–Whitney t-test. Data in (c), (d) and (j) were analyzed comparing each condition with the control (HC), by Kruskal-Wallis with uncorrected Dunn’s test; data in the remaining graphs were analyzed comparing treatments within each group by Mann–Whitney t-test; p-values are shown in the graphs. Each data point represents the data from a single patient/subject (biological replicates).

Supplementary Figure 4

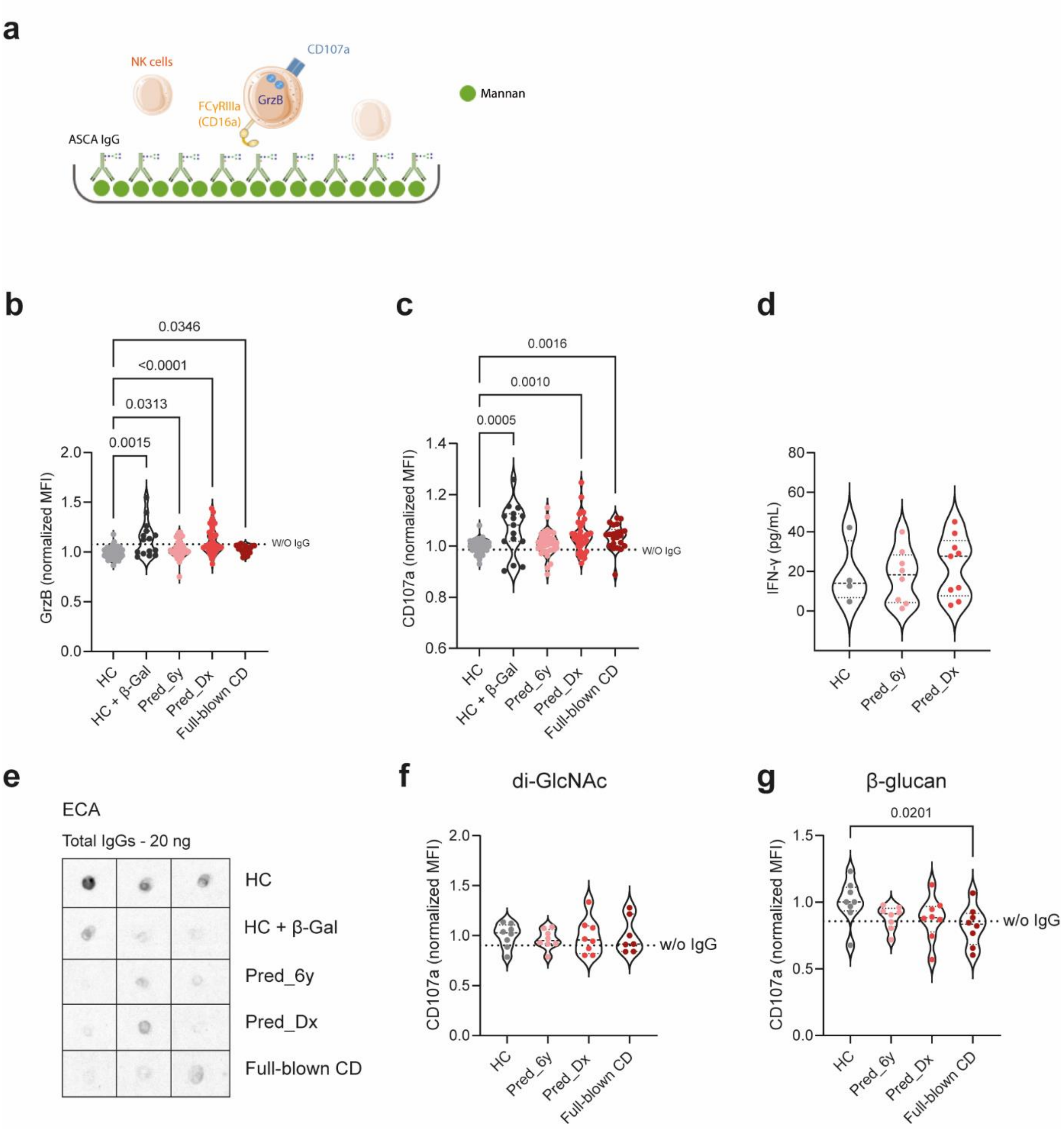

Supplementary Figure 4. NK cell activation and functional characterization upon IgG binding. (a) ASCA were isolated from total IgGs through binding to mannan-coated plates and then incubated with natural killer (NK) cells**. (b)** NK cells incubated with ASCA from preclinical (n=42), diagnosed CD (Pred_Dx, n=44; full-blown CD, n=16), or agalactosylated HC (n=15) IgGs displayed increased granzyme B production when compared with HC (n=41). **(c)** A similar profile was also found for CD107a expression (HC, n=39; HC+βGal, n=15; Pred_6y, n=33; Pred_Dx, n=37; full-blown CD, n=22). **(d)** Interferon-gamma (IFN-γ) production of NK cells when incubated with ASCA (HC, n=4; Pred_6y, n=8; Pred_Dx, n=9**). (e)** Representative dot-blot showing that total

IgGs from preclinical and diagnosed CD patients have a decreased galactosylation when compared to HC. The decrease in galactosylation of IgGs from HC treated with β-galactosidase was also confirmed. **(f-g)** CD107a expression on DCs co-cultured with diGlcNAc-specific IgGs (f) or β-glucan-specific IgGs from HC and CD patients (g) (normalized to HC); for (f), HC, n8; Pred_6y, n=8; Pred_Dx, n=8; full-blown CD, n=7. For (g), HC, n8; Pred_6y, n=8; Pred_Dx, n=8; full-blown CD, n=8. Data in (b), (c) and (d) were analyzed comparing each condition with the control (HC), by Kruskal-Wallis with uncorrected Dunn's test; data in (f) and (g) were analyzed comparing each condition with the control (HC), by one-way ANOVA with uncorrected Fisher's LSD. Each data point represents the data from a single patient/subject (biological replicates).

Supplementary Figure 5

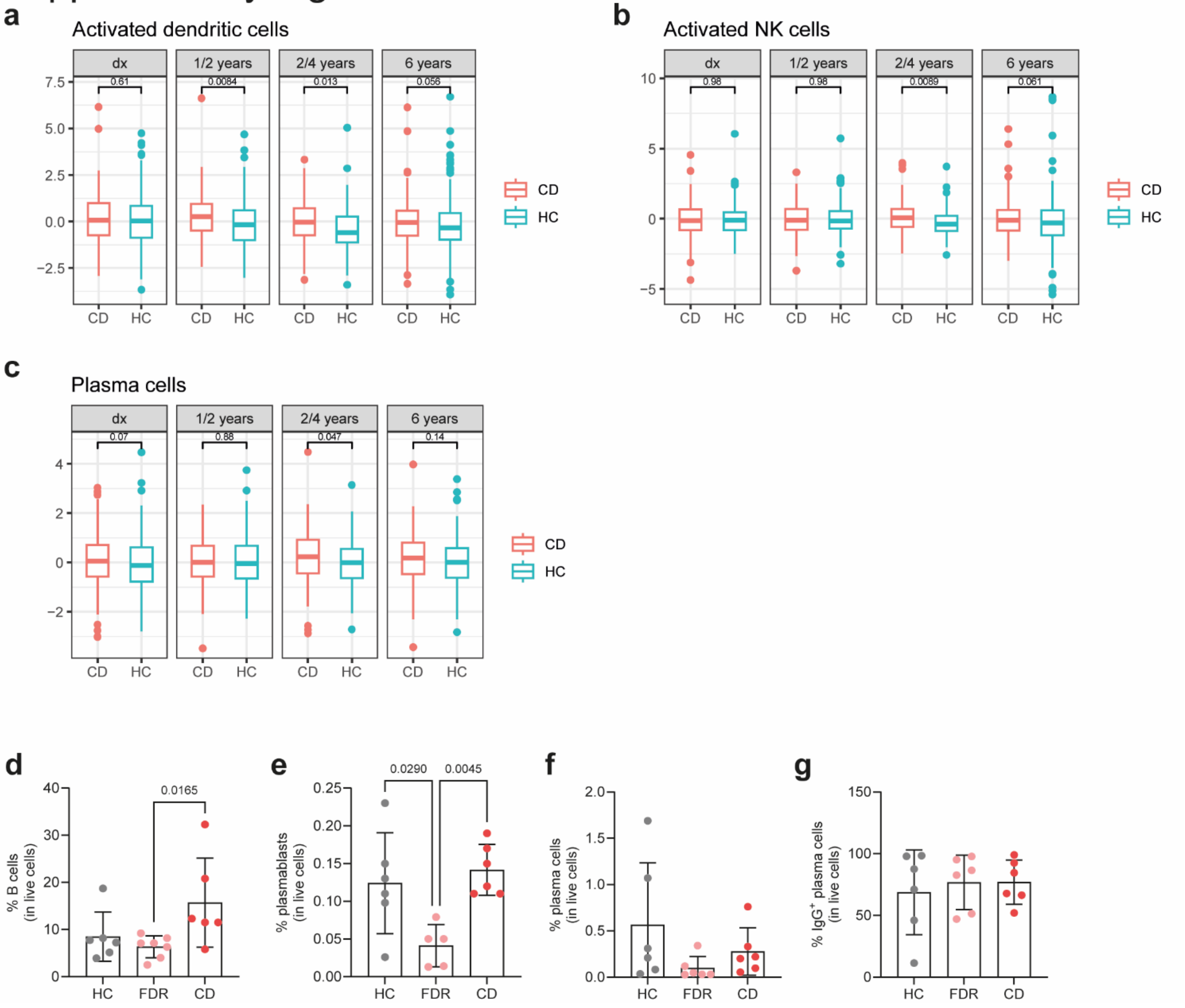

Supplementary Figure 5. Activation and functional characterization of immune cells. (a-c) Relative frequency of immune profile of activated dendritic cells, activated NK cells, and plasma cells from Crohn's disease (CD) patients versus healthy controls (HC), according to SomaLogic analysis (n=200 CD, n=200 HC). **(d-g)** PBMCs isolated from blood of healthy controls (HC), first-degree relatives from CD patients (FDR), and CD patients with inaugural disease were analyzed for the frequency of B cells (n=6 HC, n=7 FDR, n=6 CD), plasmablasts (n=6 HC, n=5 FDR, n=6 CD), and plasma cells (total and IgG^{+}; n=6 HC, n=5 FDR, n=6 CD). Data were analyzed comparing each group with FDR, by Kruskal-Wallis test with uncorrected Dunn's test, and presented as mean ± SD. Each data point represents the data from a single patient/subject (biological replicates).

Supplementary Figure 6

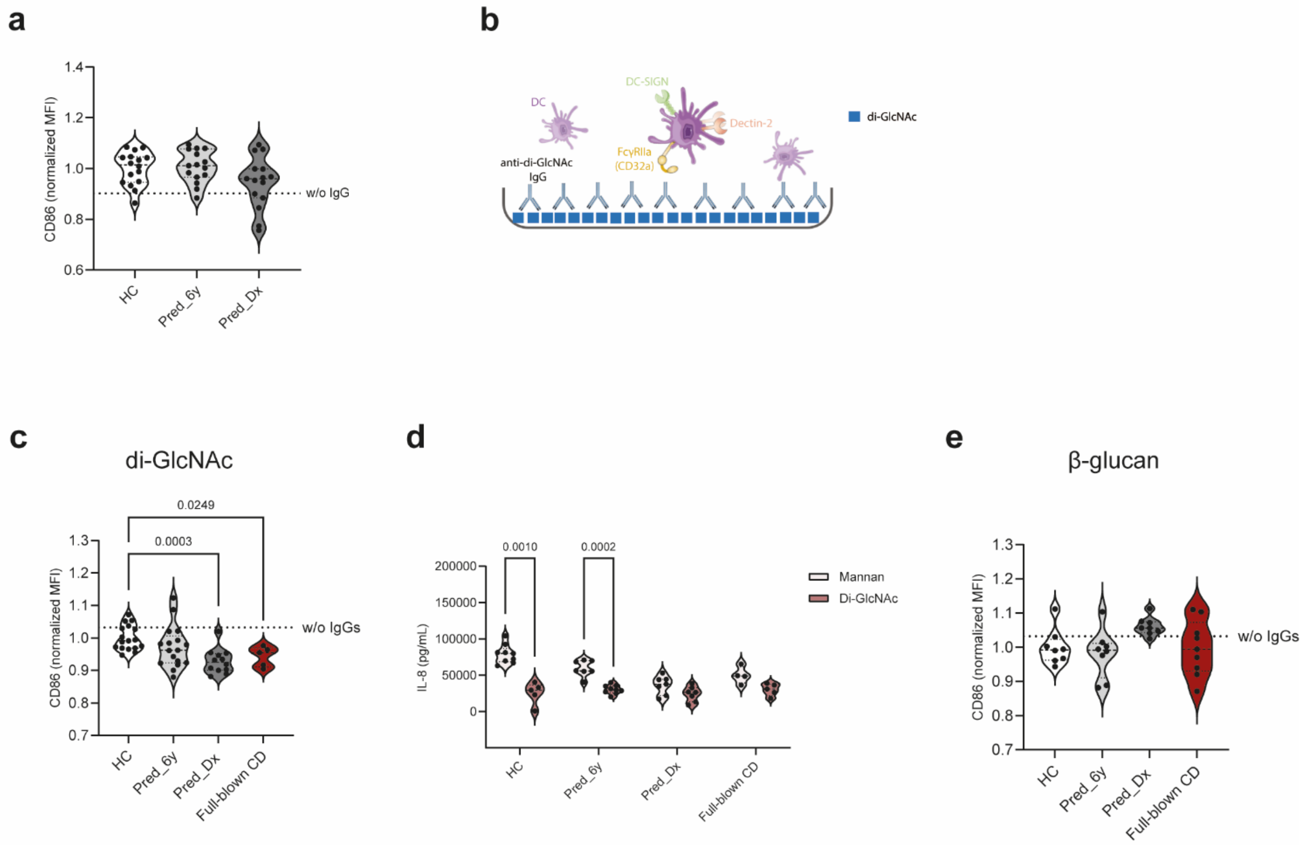

Supplementary Figure 6. Dendritic cell activation upon non-ASCA IgG incubation. (a) CD86 expression on DCs incubated with unbounded IgGs (not bound to mannan) (HC, n=16; Pred_6y, n=15; Pred_Dx, n=15). Data were analyzed by Kruskal-Wallis test. **(b)** Total IgGs were cultured on di-GlcNAc-coated plates to select anti-di-GlcNAc IgGs, and then incubated with DCs. **(c)** CD86 expression on DCs co-cultured with diGlcNAc-specific IgGs from HC and CD patients (normalized to HC); HC, n=17; Pred_6y, n=17; Pred_Dx, n=12; full-blown CD, n=5. Data were analyzed comparing each condition with the control (HC) by one-way ANOVA with uncorrected Fisher's LSD. **(d)** IL-8 production of DCs when incubated with di-GlcNAc-specific IgGs, compared to ASCA. Mannan condition is composed by n=9 HC, n=7 Pred_6y, n=7 Pred_Dx and n=4 full-blown CD; di-GlcNAc condition is composed by n=5 HC, n=9 Pred_6y, n=7 Pred_Dx and n=5 full-blown CD. Data analyzed comparing treatments within each group by Mann–Whitney t-test. **(e)** CD86 expression on DCs co-cultured with β-glucan-specific IgGs from HC and CD patients (normalized to HC); HC, n=8; Pred_6y, n=8; Pred_Dx, n=8; full-blown CD, n=9. Data were analyzed comparing each condition with the control (HC) by one-way ANOVA with uncorrected Fisher's LSD. Each data point in the figures represents the data from a single patient/subject (biological replicates).

Supplementary Figure 7

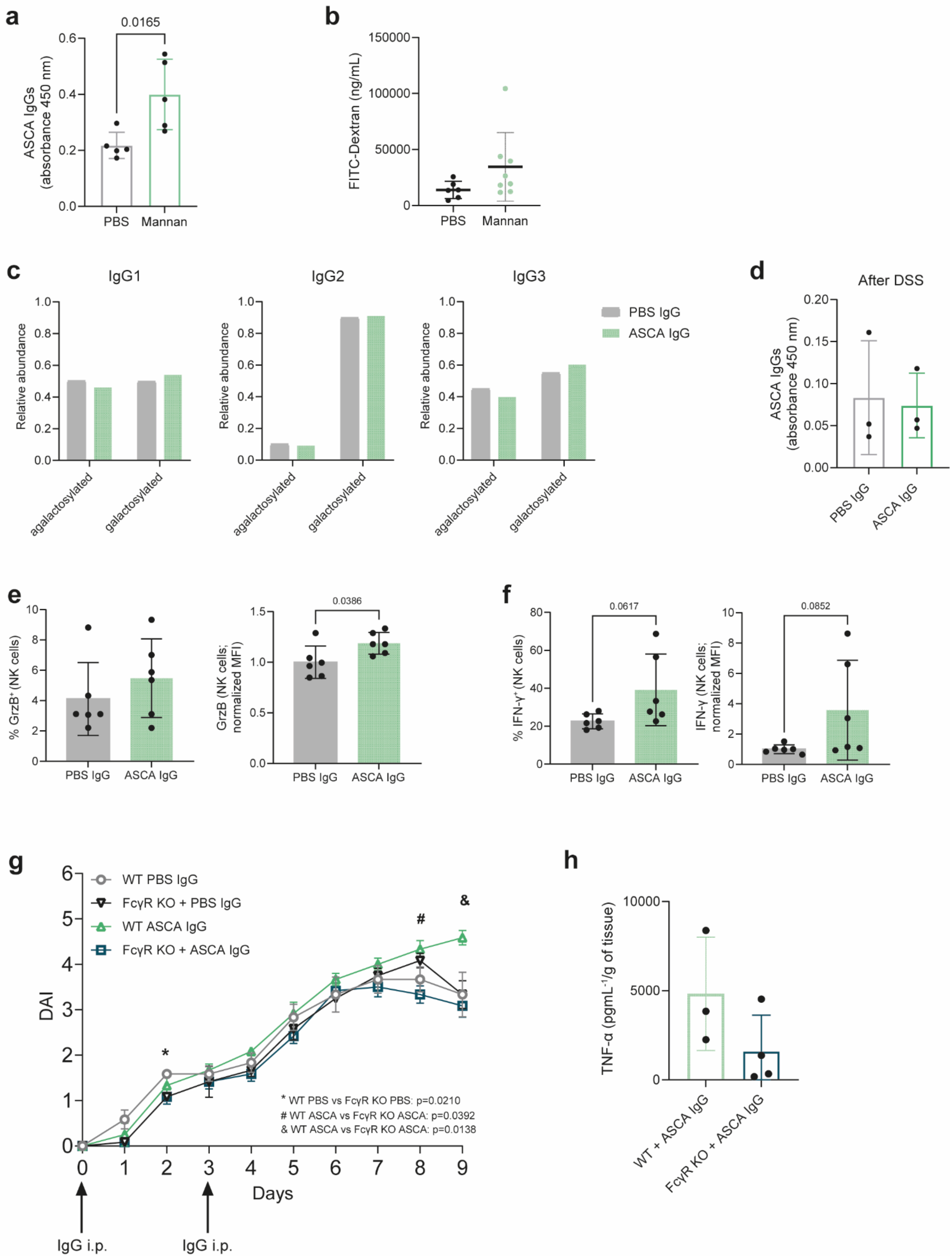

Supplementary Figure 7. Characterization of murine ASCA IgGs and impact in intestinal permeability. **(a)** Mice were inoculated with 12 mg of mannan subcutaneously, every 3 days, during 5 weeks (n=5). Control group received PBS by the same route in similar timepoints (n=5). ASCA enrichment was assessed after 5-week treatment by ELISA. **(b)** Intestinal permeability was assessed by FITC-dextran assay (PBS, n=6; mannan, n=8). **(c)** Murine ASCA galactosylation levels were assessed for IgG1, IgG2 and IgG3 isoforms in a pool of IgGs collected from several mice immunized with mannan. The galactosylation proportion was also assessed in mice treated with the vehicle (PBS). **(d)** ASCA enrichment in mice treated with ASCA IgGs (n=3) or PBS IgGs (n=3) upon DSS-induced colitis. **(e-f)** Frequency and expression of Granzyme B (e) and IFN-γ (f) in NK cells in the colon of mice immunized with ASCA (n=6) or PBS IgGs (n=6). **(g)** WT and FcγR KO mice were inoculated twice with 100 µg of total IgGs (ASCA-enriched or from PBS-injected mice - control) (n=4/group), while colitis was chemically induced by administration of 2% DSS in drinking water. WT mice inoculated with ASCA IgGs displayed increased susceptibility. * represents significant differences between WT PBS vs FcγR KO PBS (day 2 post-DSS); # and & represent significant differences between WT ASCA vs FcγR KO ASCA (day 8 and day 9 post-DSS, respectively). **(h)** TNF-α was quantified in supernatants from colonic extracts of WT ASCA IgG (n=3) and FcγR KO ASCA IgG (n=4). In (g), data were analyzed by two-way ANOVA with Tukey's multiple comparison test and are presented as mean ± SEM. Scatter dot plots are presented as mean ± SD. Frequency of Granzyme B in (e) and data in (h) were analyzed by two-tailed Mann-Whitney test. The remaining were analyzed by two-tailed unpaired t-test. Each data point in the figures represents the data from a single subject (biological replicates).

Supplementary Figure 8

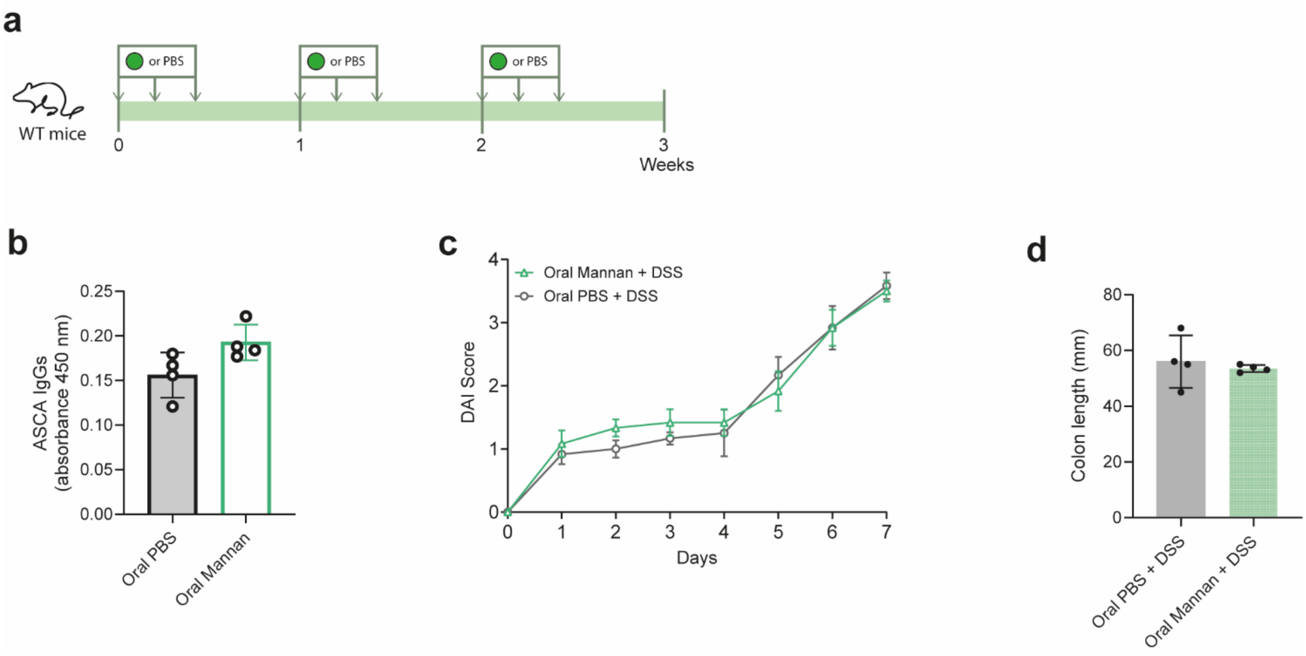

Supplementary Figure 8. Anti-mannan IgGs derived from oral supplementation with mannan do not impact colitis susceptibility. (a) Wild-type (WT) mice were treated with mannan by oral gavage to promote the generation of anti-mannan antibodies (ASCA-like) (n=4). Control mice were treated with PBS (n=4). **(b)** Anti-mannan IgGs were quantified in the serum and collected from both groups (n=4/group). **(c-d)** Colitis was chemically induced by administration of 2% DSS in drinking water. No major alterations were found for colitis susceptibility, as shown by a similar DAI (c), and no alterations in colon length (d) (n=4/group). Data in (c) were analyzed by two-way ANOVA with Šídák post-test and are presented as mean ± SEM. Data in (b) and (c) were analyzed by two-tailed unpaired t-test and are presented as mean ± SD. Each data point in the figures represents the data from a single subject (biological replicates).

Supplementary Figure 9

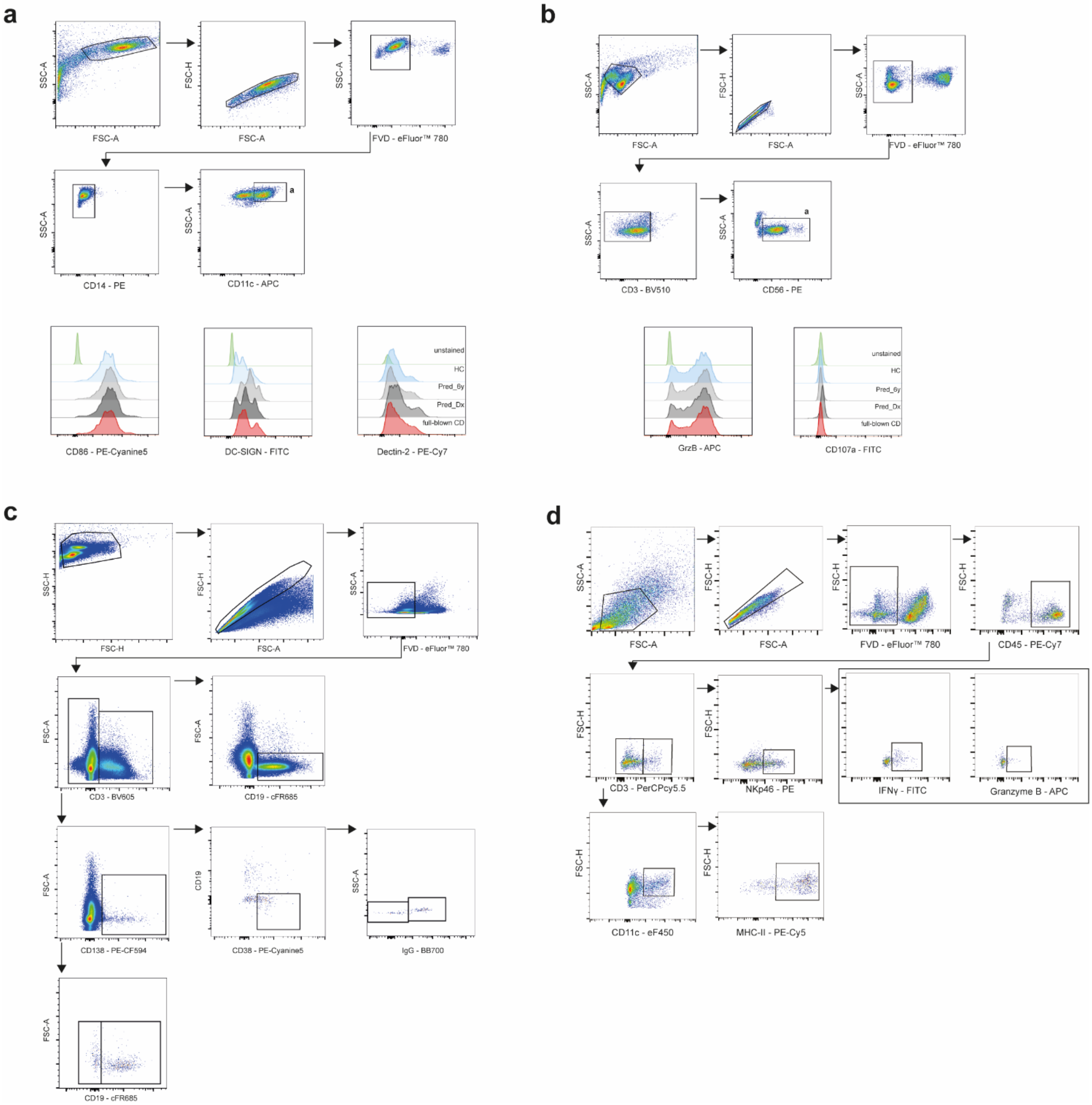

Supplementary Figure 9. Gating strategies used in the flow cytometry analyses. (a) For the co-culture of human DCs with ASCA IgGs, cells were gated by CD14low, CD11c+ cells, after exclusion of duplets and dead cells. The median fluorescence intensity (MFI) of CD86, DC-SIGN and dectin-2 was assessed in CD11c+ cells. **(b)** For the co-culture of human NK cells with ASCA IgGs, cells were identified as CD3-, CD56+ cells, after exclusion of duplets and dead cells. The median fluorescence intensity (MFI) of granzyme B and CD107a (LAMP-1) was assessed in CD3-CD56+ cells. **(c)** For the analysis of B cells, plasma cells and plasmablasts, duplets and dead cells were excluded and cell populations were defined as follows: B cells - CD3-CD19+;

plasma cells - CD3-CD138+CD19-CD38+; plasmablasts - CD3-CD138+CD19+. **(d)** For the animal model, duplets and dead cells were excluded, and DCs were identified as CD45+CD3-CD11c+ cells, while NK cells were selected as CD45+CD3-NKp46+ cells. IFNγ and granzyme B were assessed within NK cells, while MHC-II was analysed within DCs.

Figure 4e – unprocessed blot

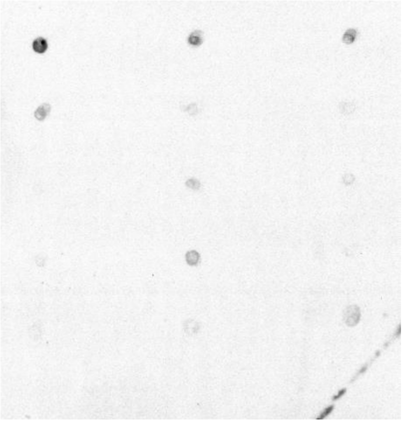

Supplement: Supplementary file 1 — Supplementary Methods, Tables 1–7 and Figs. 1–9. [file 41590_2024_1916_MOESM1_ESM.pdf]
