# Supplementary material for: A unique serum IgG glycosylation signature predicts development of Crohn’s disease and is associated with pathogenic antibodies to mannose glycan
Source: Nat Immunol. 2024 Jul 30;25(9):1692–703. doi: 10.1038/s41590-024-01916-8 (PMC11362009; doi:10.1038/s41590-024-01916-8)
Supplement: Supplementary file 2 — Reporting Summary [file 41590_2024_1916_MOESM2_ESM.pdf]

nature portfolio

Corresponding author(s): Salomé S. Pinho

Last updated by author(s): Jun 20, 2024

Reporting Summary

Nature Portfolio wishes to improve the reproducibility of the work that we publish. This form provides structure for consistency and transparency in reporting. For further information on Nature Portfolio policies, see our Editorial Policies and the Editorial Policy Checklist.

Statistics

For all statistical analyses, confirm that the following items are present in the figure legend, table legend, main text, or Methods section.

n/a	Confirmed	
☐	☒	The exact sample size (*n*) for each experimental group/condition, given as a discrete number and unit of measurement
☐	☒	A statement on whether measurements were taken from distinct samples or whether the same sample was measured repeatedly
☐	☒	The statistical test(s) used AND whether they are one- or two-sided<br>*Only common tests should be described solely by name; describe more complex techniques in the Methods section.*
☐	☒	A description of all covariates tested
☐	☒	A description of any assumptions or corrections, such as tests of normality and adjustment for multiple comparisons
☐	☒	A full description of the statistical parameters including central tendency (e.g. means) or other basic estimates (e.g. regression coefficient) AND variation (e.g. standard deviation) or associated estimates of uncertainty (e.g. confidence intervals)
☐	☒	For null hypothesis testing, the test statistic (e.g. *F*, *t*, *r*) with confidence intervals, effect sizes, degrees of freedom and *P* value noted<br>*Give P values as exact values whenever suitable.*
☒	☐	For Bayesian analysis, information on the choice of priors and Markov chain Monte Carlo settings
☒	☐	For hierarchical and complex designs, identification of the appropriate level for tests and full reporting of outcomes
☒	☐	Estimates of effect sizes (e.g. Cohen's *d*, Pearson's *r*), indicating how they were calculated

Our web collection on statistics for biologists contains articles on many of the points above.

Software and code

Policy information about availability of computer code

Data collection	FACSDiva™ V6.1.3 software; SpectraFlow v3.2.1; MassLynx software 4.1; HyStar software version 4.1
Data analysis	FlowJo version 10.5.3; FeatureCounts (v1.5.0-p3); R statistical software (version 3.6.3 and v4.1.2); DESeq2 (v1.34); apeglm (v1.23.1); clusterProfiler (v4.11.0.001); complexHeatmap (v2.10.0); ggplot2 (v3.4.0); GraphPad Prism 10.2.3

For manuscripts utilizing custom algorithms or software that are central to the research but not yet described in published literature, software must be made available to editors and reviewers. We strongly encourage code deposition in a community repository (e.g. GitHub). See the Nature Portfolio guidelines for submitting code & software for further information.

Data

Policy information about availability of data

All manuscripts must include a data availability statement. This statement should provide the following information, where applicable:

- Accession codes, unique identifiers, or web links for publicly available datasets
- A description of any restrictions on data availability
- For clinical datasets or third party data, please ensure that the statement adheres to our policy

The RNA sequencing data have been deposited in the EMBL Nucleotide Sequence Database – European Nucleotide Archive (ENA) with the dataset identifier PRJEB76671. Patients-related data may be subject to confidentiality. Data displayed in Figure 1, Supplementary Figures 1a, 1b and 1c, Supplementary Figure 2, and

Supplementary Figures 5a, 5b and 5c are available from the corresponding author upon request. The source data for remaining Figures and Supplementary Figures are provided as a Source Data file. Reagents, materials and protocols are available upon request to the corresponding author.

Research involving human participants, their data, or biological material

Policy information about studies with human participants or human data. See also policy information about sex, gender (identity/presentation), and sexual orientation and race, ethnicity and racism.

Reporting on sex and gender	We use the term "sex" consistently throughout the manuscript. All information regarding donors is listed on supplementary table 1, 3 and 4.
Reporting on race, ethnicity, or other socially relevant groupings	IgG glycome analysis was performed by two-sided t-test from logistic regression model (-log10 scale) after adjusting for sex, race and age.
Population characteristics	For the PREDICTS cohort, a total of 750 individuals were recruited (CD: n=251, 81.64% males; HC: n=250, 91.68% males; UC: n=249, 89.72% males), comprising a total of 2991 serum samples. The mean age was: CD-31.70 years; HC-29.67 years; UC-29.77 years.<br>The established CD cohort was composed by 10 individuals (70% males) comprising 33 serum samples. The mean age was 53.50 years, and the mean time since diagnosis was 19.04 years. Information on clinical activity or treatments is presented in supplementary information.<br>The cohort for first-degree relatives (FDR) and inaugural CD patients was composed by: 6 inaugural CD (83% male); 7 FDR (29% male) and 6 healthy control individuals (17% males). The mean age was 32 years for inaugural CD, 26 years for FDR and 27 for healthy individuals.
Recruitment	Samples were retrieved from the US Department of Defense Serum Repository (DoDSR) or recruited in the local hospitals. Only patients able to give their informed consent were recruited. We cannot identify any specific bias in the process that may significantly influence the results.
Ethics oversight	This study was approved as non-human subjects' research (PJT 19-08) by the Naval Medical Research Command, Silver Spring, MD. Ethical approval was obtained at the Ethical Committees of all Hospitals.

Note that full information on the approval of the study protocol must also be provided in the manuscript.

Field-specific reporting

Please select the one below that is the best fit for your research. If you are not sure, read the appropriate sections before making your selection.

☒ Life sciences ☐ Behavioural & social sciences ☐ Ecological, evolutionary & environmental sciences

For a reference copy of the document with all sections, see nature.com/documents/nr-reporting-summary-flat.pdf

Life sciences study design

All studies must disclose on these points even when the disclosure is negative.

Sample size	This study is focused on the exploitation of altered immune-mediated mechanisms in the context of health to inflammation transition. We believe that the final sample size is enough considering that all sub-groups of age,sex, disease severity are represented by a reasonable number of individuals and conditions, as well as based on previous evidence. By adding a control group with a significant dimension, we also add reliability to our results.
Data exclusions	No data were excluded from the analysis.
Replication	All the assays involved testing independent clinical samples from patients and controls. For animal experimentation, biological replicates were considered in the analysis. The assay with BMDCs was performed once, but with four technical replicates.
Randomization	Randomization was taken into consideration in serum IgG glycome profile. The groups for comparison were established according to the clinical status of the patients/individuals, and the analysis was performed on that basis.
Blinding	The investigators were blinded to group allocation during data collection and analysis. A code was assigned to each participant and the data collection and analysis was performed without the knowledge of which group they appertain.

Reporting for specific materials, systems and methods

We require information from authors about some types of materials, experimental systems and methods used in many studies. Here, indicate whether each material, system or method listed is relevant to your study. If you are not sure if a list item applies to your research, read the appropriate section before selecting a response.

Materials & experimental systems

n/a	Involved in the study	
☐	☒	Antibodies
☒	☐	Eukaryotic cell lines
☒	☐	Palaeontology and archaeology
☐	☒	Animals and other organisms
☒	☐	Clinical data
☒	☐	Dual use research of concern
☐	☐	Plants

Methods

n/a	Involved in the study	
☒	☐	ChIP-seq
☐	☒	Flow cytometry
☒	☐	MRI-based neuroimaging

Antibodies

Antibodies used

1. Brilliant Violet 510 anti-human CD3, clone OKT3, 317332, BioLegend
2. PE anti-human CD56, clone HCD56, 318306, BioLegend
3. APC anti-human Granzyme B, MHGB05, Invitrogen
4. FITC anti-human CD107a (LAMP-1), 328606, BioLegend
5. PE anti-human CD14, clone 61D3, 12-0149-42, Invitrogen
6. APC anti-human CD11c, clone B015, 17-0128-42, Invitrogen
7. PE-Cyanine5 anti-human CD86, clone IT2.2, 15-0869-42, Invitrogen
8. Rabbit anti-human CD209, AHP627, Bio-Rad
9. Polyclonal Swine anti rabbit Immunoglobulins/FITC, F0205, Dako
10. Anti-hDectin2 Affinity Purified Goat IgG, AF3114, R&D Systems
11. Polyclonal Rabbit anti-Goat Immunoglobulins/Biotinylated, E0466, Dako
12. Streptavidin PE-Cyanine7, 25-4317-82, Invitrogen
13. PE-CF594 anti-human CD138, clone MI15, 564606, BD Biosciences
14. PE-Cyanine5 anti-human CD38, clone HIT2, 303507, BioLegend
15. BV605 anti-human CD3, clone 17A2, 100237, BioLegend
16. cFR685 anti-human CD19, clone HIB19, R7-20118, Cytek
17. BB700 anti-human IgG, clone G18-145, 742235, BD Biosciences
18. PE-Cyanine7 anti-human IgM, clone MHM-88, 314531, BioLegend
19. BV605 anti-mouse CD45, clone 30-F11, 103139, BioLegend
20. eF450 anti-mouse CD11c, clone N418, 48-0114-82, Invitrogen
21. PE anti-mouse NKp46, clone 29A1.4, 12-3351-80, Invitrogen
22. PerCP-Cy5.5 anti-mouse CD3, 100328, BioLegend
23. PE-Cy5 anti-mouse MHCII, clone M5/114.15.2, 15-5321-81, Invitrogen
24. PE-Cy7 anti-mouse CD45, clone 30-F11, 25-0451-82, Invitrogen
25. FITC anti-mouse IFNg, clone XMG1.2, 11-7311-82, Invitrogen
26. PE-Cy7 anti-mouse CD16/32, clone 93, 101318, BioLegend
27. Purified anti-mouse CD16/32, clone 93, 101302, BioLegend

Validation

1. https://www.biolegend.com/en-us/products/brilliant-violet-510-anti-human-cd3-antibody-8009
2. https://www.biolegend.com/en-us/products/pe-anti-human-cd56-ncam-antibody-3796
3. https://www.thermofisher.com/antibody/product/Granzyme-B-Antibody-clone-GB12-Monoclonal/MHGB05
4. https://www.biolegend.com/en-us/products/fitc-anti-human-cd107a-lamp-1-antibody-4966
5. https://www.thermofisher.com/antibody/product/CD14-Antibody-clone-61D3-Monoclonal/12-0149-42
6. https://www.thermofisher.com/antibody/product/CD11c-Antibody-clone-BU15-Monoclonal/17-0128-42
7. https://www.thermofisher.com/antibody/product/CD86-B7-2-Antibody-clone-IT2-2-Monoclonal/15-0869-42
8. https://www.bio-rad-antibodies.com/polyclonal/human-cd209-antibody-ahp627.html?f=purified
9. https://www.agilent.com/store/pt_BR/Prod-F020502-2/F020502-2
10. https://www.rndsystems.com/products/human-dectin-2-clec6a-antibody_af3114
11. https://www.agilent.com/en/oem-polyclonal-antibodies
12. https://www.thermofisher.com/order/catalog/product/25-4317-82
13. https://www.bdbiosciences.com/en-au/products/reagents/flow-cytometry-reagents/research-reagents/single-color-antibodies-ruo/pe-cf594-mouse-anti-human-cd138.564606
14. https://www.biolegend.com/en-us/products/pe-cyanine5-anti-human-cd38-antibody-747
15. https://www.biolegend.com/en-us/products/brilliant-violet-605-anti-mouse-cd3-antibody-8503
16. https://welcome.cytekbio.com/hubfs/TDS%20cFluor%20Reagents/R8-50117%20Rev.B_TDS_cFluor%20R685%20huCD19%20(HIB19).pdf
17. https://www.bdbiosciences.com/en-ie/products/reagents/flow-cytometry-reagents/research-reagents/single-color-antibodies-ruo/bb700-mouse-anti-human-igg.742235
18. https://www.biolegend.com/en-us/products/pe-cyanine7-anti-human-igm-antibody-12467
19. https://www.biolegend.com/en-us/products/brilliant-violet-605-anti-mouse-cd45-antibody-8721
20. https://www.thermofisher.com/antibody/product/CD11c-Antibody-clone-N418-Monoclonal/48-0114-82
21. https://www.thermofisher.com/antibody/product/CD335-NKp46-Antibody-clone-29A1-4-Monoclonal/12-3351-80
22. https://www.biolegend.com/en-us/products/percp-cyanine5-5-anti-mouse-cd3epsilon-antibody-4191
23. https://www.thermofisher.com/antibody/product/MHC-Class-II-I-A-I-E-Antibody-clone-M5-114-15-2-Monoclonal/15-5321-81
24. https://www.thermofisher.com/antibody/product/CD45-Antibody-clone-30-F11-Monoclonal/25-0451-82
25. https://www.thermofisher.com/antibody/product/IFN-gamma-Antibody-clone-XMG1-2-Monoclonal/11-7311-82

26. https://www.biolegend.com/en-us/products/pe-cyanine7-anti-mouse-cd16-32-antibody-6355
27. https://www.biolegend.com/en-us/products/purified-anti-mouse-cd16-32-antibody-190

Animals and other research organisms

Policy information about studies involving animals; ARRIVE guidelines recommended for reporting animal research, and Sex and Gender in Research

Laboratory animals	C57BL/6 were used as WT mice. FcgR KO mice (C57BL/6 background) were kindly provided by Jeffrey V. Ravetch (Rockefeller University, New York, USA) and Mark S. Cragg (University of Southampton, United Kingdom). Mice were housed at the AAALAC-accredited i3S animal facility in a controlled-temperature room (20-24ºC), 45-55% humidity, and under a 12-hour light/12-hour dark period. WT mice were used between 6-13 weeks of age. FcgR KO mice were used between 10-13 weeks of age.
Wild animals	No wild animals were used in the study.
Reporting on sex	For mannan inoculation and collection, both female and male mice were used. Since the goal was to collect mannan-specific IgGs (or the respective PBS IgGs from the control), sex is not expected to act as a confounding factor. For DSS experiments only with WT animals (Fig 4b), females were used to reduce possible sex-dependent variability, taking into consideration our previous evidence. For experiments with FcgR KO mice, both males and females were used; aged- and sex-matched WT were used as controls; no major variability was found.
Field-collected samples	N/A
Ethics oversight	All mouse procedures were approved by the Institute for Research and Innovation in Health (i3S) Animal Ethics Committee for animal experimentation under Portuguese regulations (DGAV license number 009268/2022-06-02).

Note that full information on the approval of the study protocol must also be provided in the manuscript.

Plants

Seed stocks	N/A
Novel plant genotypes	N/A
Authentication	N/A

Flow Cytometry

Plots

Confirm that:

☒ The axis labels state the marker and fluorochrome used (e.g. CD4-FITC).

☒ The axis scales are clearly visible. Include numbers along axes only for bottom left plot of group (a 'group' is an analysis of identical markers).

☒ All plots are contour plots with outliers or pseudocolor plots.

☒ A numerical value for number of cells or percentage (with statistics) is provided.

Methodology

Sample preparation	PMBCs were obtained from buffy coats from healthy donors, as well as peripheral blood from inaugural CD patients (collected at the time of diagnosis) and first-degree relatives of CD patients. Anticoagulated blood was diluted in PBS (ratio 1:1), slowly layered onto Lymphoprep™ (ratio 1:2) and centrifuged by gradient at 900 x g for 30 minutes at room temperature. After centrifugation, the interface containing PBMCs was transferred to a conical centrifuge tube and washed with PBS. PBMCs from inaugural CD patients (collected at the time of diagnosis), first-degree relatives of CD patients, and healthy controls were collected and the plasma cell characterization was performed by flow cytometry, using surface and intracellular markers. Cells were fixed and permeabilized with Transcription Factor Staining Buffer Set (eBioscience), followed by intracellular staining.<br>For DC culture, after 6h cells were washed with FACS buffer and stained for viability and surface markers. All incubations were performed for 30 minutes at 4°C. Samples were fixed by 2% of paraformaldehyde (Sigma-Aldrich).<br>For NK culture, following 1 hour of culture, 10 µg/ml of Brefeldin A and GolgiStop™ Protein Transport Inhibitor were added and incubated 5 hours in similar conditions. After incubation, cells were stained for viability, as well as for surface markers. Cells were then fixed and permeabilized with Transcription Factor Staining Buffer Set (eBioscience), followed by intracellular

staining.
In in vivo experiments, lamina propria leukocytes were isolated from colonic fragments incubated in DMEM medium supplemented with 1mM $CaCl_2$, 1mM $MgCl_2$, 1.5 mg/mL of collagenase IV (Sigma), and 0.4 mg/mL of dispase (Gibco), under 100 rpm agitation at 37ºC for 40 minutes. Tissues were dissociated and filtered through a 70 µm cell strainer (BD Biosciences). Cell suspension was resuspended in RPMI 1640 medium supplemented with 10% FBS, and 1% penicillin/streptomycin, and layered upon Lymphoprep solution in a proportion of 1:2 (Lymphoprep:cell suspension). After gradient centrifugation at 800g for 20 minutes at 20ºC (without acceleration or break), immune cells (retained in the interface) were collected for the staining.

Instrument

FACSCanto™ II system (BD Biosciences); Cytek Aurora™ system (Cytek).

Software

Samples were acquired using the BD FACSDiva software V6.1.3 or SpectraFlow v3.2.1, and data was analyzed using FlowJo software, version 10.5.3.

Cell population abundance

No cell sorting was performed in this study.

Gating strategy

For the co-culture of human DCs with ASCA IgGs, cells were gated by CD14low, CD11c+ cells, after exclusion of duplets and dead cells. The median fluorescence intensity (MFI) of CD86, DC-SIGN and dectin-2 was assessed in CD11c+ cells.
For the co-culture of human NK cells with ASCA IgGs, cells were identified as CD3-, CD56+ cells, after exclusion of duplets and dead cells. The median fluorescence intensity (MFI) of granzyme B and CD107a (LAMP-1) was assessed in CD3-CD56+ cells.
For the analysis of B cells, plasma cells and plasmablasts, duplets and dead cells were excluded and cell populations were defined as follows: B cells - CD3-CD19+; plasma cells - CD3-CD138+CD19-CD38+; plasmablasts - CD3-CD138+CD19+.
For the animal model, duplets and dead cells were excluded, and DCs were identified as CD45+CD3-CD11c+ cells, while NK cells were selected as CD45+CD3-NKp46+ cells. IFNγ and granzyme B were assessed within NK cells, while MHC-II was analysed within DCs. The gating strategy is included in the manuscript in the Supplementary Information.

☒ Tick this box to confirm that a figure exemplifying the gating strategy is provided in the Supplementary Information.
